# Saliency Detection Based on Multiple-Level Feature Learning

**DOI:** 10.3390/e26050383

**Published:** 2024-04-30

**Authors:** Xiaoli Li, Yunpeng Liu, Huaici Zhao

**Affiliations:** 1 Shenyang Institute of Automation, Chinese Academy of Sciences, Shenyang 110169, China; ypliu@sia.cn (Y.L.); hczhao@sia.cn (H.Z.); 2 Institutes for Robotics and Intelligent Manufacturing, Chinese Academy of Sciences, Shenyang 110169, China; 3 University of Chinese Academy of Sciences, Beijing 100049, China; 4 Key Laboratory of Opto-Electronic Information Processing, Shenyang 110169, China; 5 The Key Lab of Image Understanding and Computer Vision, Shenyang 110619, China; 6 School of Computer Science and Engineering, Shenyang Jianzhu University, Shenyang 110168, China

**Keywords:** saliency detection, feature learning, deep neural network (DNN)

## Abstract

Finding the most interesting areas of an image is the aim of saliency detection. Conventional methods based on low-level features rely on biological cues like texture and color. These methods, however, have trouble with processing complicated or low-contrast images. In this paper, we introduce a deep neural network-based saliency detection method. First, using semantic segmentation, we construct a pixel-level model that gives each pixel a saliency value depending on its semantic category. Next, we create a region feature model by combining both hand-crafted and deep features, which extracts and fuses the local and global information of each superpixel region. Third, we combine the results from the previous two steps, along with the over-segmented superpixel images and the original images, to construct a multi-level feature model. We feed the model into a deep convolutional network, which generates the final saliency map by learning to integrate the macro and micro information based on the pixels and superpixels. We assess our method on five benchmark datasets and contrast it against 14 state-of-the-art saliency detection algorithms. According to the experimental results, our method performs better than the other methods in terms of F-measure, precision, recall, and runtime. Additionally, we analyze the limitations of our method and propose potential future developments.

## 1. Introduction

Humans can quickly find the regions that capture their attention in any scene. Therefore, how to enable computers to perform this task in an unsupervised manner has become an important problem. Saliency detection methods aim to produce saliency maps for an image. Various tasks have employed saliency detection, such as visual tracking [1], image segmentation [2,3,4], and object recognition [5]. These saliency detection methods fall into the following two groups: top-down and bottom-up. In order to compute saliency maps, the bottom-up methods rely on low-level features like color, brightness, texture, or position. To improve the detection accuracy, they frequently make use of prior knowledge, such as center prior [6], border prior [7], and color prior [8]. A combination of multi-scale color, intensity, and orientation saliency maps was first proposed by Itti et al. [9]. This method only focuses on the local information of the salient regions, ignoring the global information, and thus has a poor detection performance. Subsequently, methods using contrast calculation, including local contrast or global contrast, were widely applied to saliency detection. Achanta et al. [10] represented the saliency of the image by calculating the difference between the average color feature of the entire image and the color feature of each individual pixel. Even though this algorithm is easy to use and effective, the detection results contain a lot of background noise. Later, some scholars employed the boundary area of an image as a prior knowledge for saliency detection, because compared with the background region, the foreground region is rarely located at the image boundary. Jiang et al. [11] constructed an absorbing Markov chain, taking the background region as absorbing nodes, and calculated the transition times from other nodes to absorbing nodes as their saliency values. A graph-based manifold ranking saliency detection method (MR) was presented by Yang et al. [12], taking the background region as query nodes, ranking other nodes with query nodes, and calculating the saliency of each node. Li et al. [13] reconstructed and calculated the corresponding dense reconstruction error and sparse reconstruction error to represent the saliency value.

**Figure 1:**
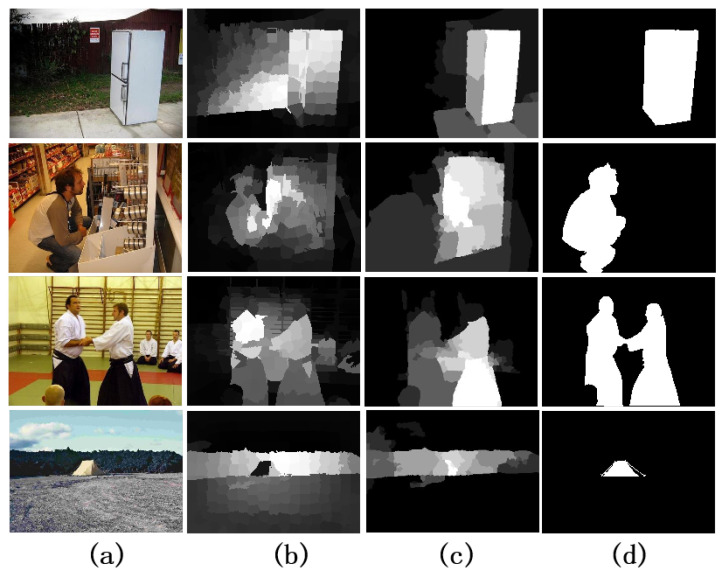
Visual comparisons of results. (**a**) Input images. (**b**) Saliency maps based on a traditional method [12]. (**c**) Saliency maps based on a DNN method [14]. (**d**) Ground truth.

But because there were no data training and learning, these methods based on low-level features do not perform as well as the detection methods based on top-down, as shown in Figure 1. The method [14] applied the classification feature of a deep neural network (DNN) to construct dense and sparse labeling maps, respectively, and put the labeling maps into the DNN to produce the final saliency map. It achieves the better detection results than the MR method [12]. The research on the top-down methods has received a lot of attention lately. Typically, top-down methods are task-driven, requiring a machine learning scheme to integrate high-level features into the process that was initially limited to specific objects or hypotheses. DNN is one of them; it outperforms conventional techniques and has demonstrated outstanding performance in computer vision tasks. As a task-driven learning method, DNN can automatically learn the optimal image representation features and classify from a huge amount of training data. The excellent performance of DNN comes from its deep structure; that is, each layer of features is a nonlinear transformation of the previous layer of features. The network’s capacity for nonlinear fitting will get stronger and stronger as the number of layers rises, and the feature extraction ability will also become stronger and stronger, thus being able to extract image features with high semantic information and discriminative performance. The typical methods are as follows: He et al. [15] designed two superpixel input sequences, and used two one-dimensional convolutional neural networks (CNNs) to combine contextual superpixel information to compute the saliency value of each superpixel. Wang et al. [16] used an encoder–decoder network in a recurrent architecture to iteratively improve the saliency map.

We propose a new saliency detection algorithm via multi-level feature learning that takes advantage of deep neural networks’ capacity to compute saliency values at both the pixel and superpixel levels. First, we feed the original input image into a DNN model, which generates a coarse contour saliency map at the pixel level. Second, we segment the original image using the simple linear iterative clustering (SLIC) method [17] and extract the high-level and low-level features of each superpixel independently. We apply the manifold ranking method [12] to compute saliency values to each superpixel unit, and obtain a superpixel-wise initial saliency prediction. Third, we take the saliency maps from the previous two steps, along with the initial image and the over-segmented image, to form an indication model, and put the model into the final deep convolutional network.

The following novel contributions are provided by this work:

(1) We propose a multi-level feature learning-based saliency detection approach that outperforms both the top-down and bottom-up contrast techniques;

(2) The superpixel-wise initial saliency prediction is refined, the salient objects are highlighted, and the background is suppressed by applying a manifold-space ranking method;

(3) In the second step, we leverage the complementary relationship of hand-crafted and deep features, and employ multi-dimension features to represent the image units more accurately;

(4) The two coarse saliency maps generated by the first and second steps can accurately locate salient objects, but some salient objects do not have sharp edges and a smooth interior. In the final step, we put the initial RGB image, superpixel image, and the two coarse saliency maps into a DNN model, using the superpixel indicator channel to accurately represent the superpixels to be classified, and obtain the final more accurate saliency map.

We have included the following portions in this paper: Section 2 discusses the relevant work. We outline our suggested procedure in Section 3. In Section 4, our experimental results are displayed in detail. In Section 5, our method’s failure cases are illustrated. In Section 6, we provide a summary of our paper.

## 2. Related Works

The cores of our method are composed of the following two aspects: manifold space ranking method [12] and DNN-based method.

### 2.1. Manifold Space Ranking Method

Manifold ranking is a subfield of semi-supervised learning classification, which is also referred to as semi-supervised regression. To put it simply, manifold ranking is the process of using positive or negative samples to determine how similar a sample is to other positive or negative samples based on the size of the ranking value. The sample is then classified based on this similarity. By using the graph model to illustrate the link between the data, the graph-based manifold ranking algorithm improves the readability of the data representation.

#### 2.1.1. Superpixel Segmentation

The manifold ranking method is based on superpixels. It uses the SLIC (simple linear iterative clustering) algorithm [17] to perform superpixel segmentation, which are groups of pixels with adjacent and similar features in terms of color and texture. Therefore, a high number of pixels can be replaced out for a small number of superpixels, which will simplify further image processing.

The SLIC algorithm first needs to converted the color space of the image from the RGB color space to the CIE-Lab color space. Each pixel corresponds to a 5-dimensional vector V[L,a,b,x,y], where (L,a,b) represents the color values and (x,y) represents the coordinates. The similarity between two pixels can be measured by their vector distance, where a greater distance implies a lower similarity.

**Figure 2:**
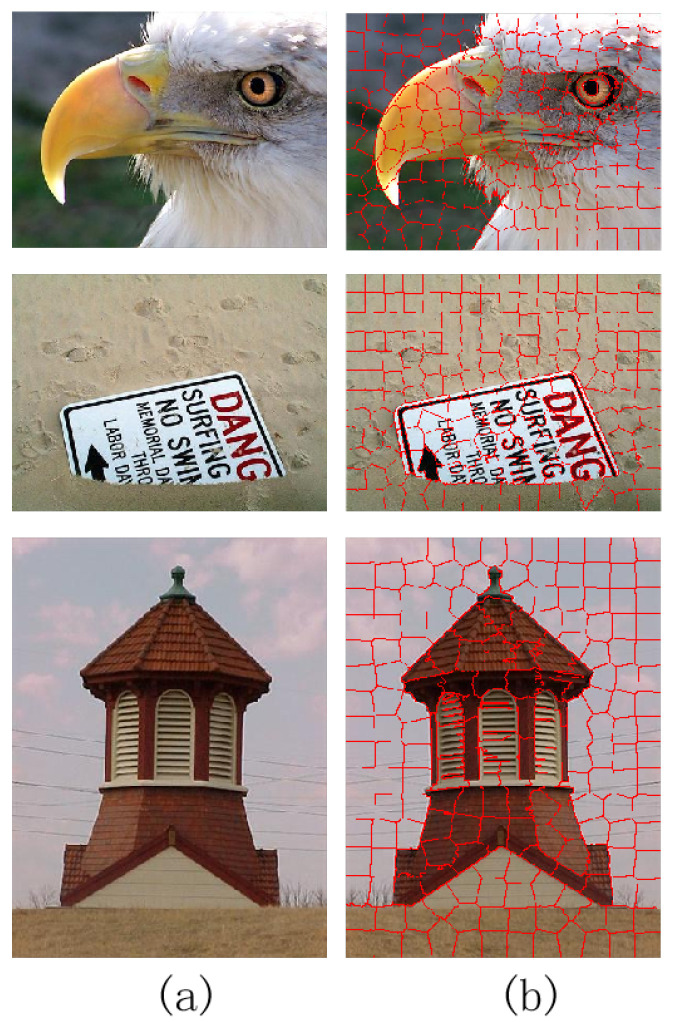
Segmented superpixels visual maps by SLIC methods (**a**) Input images. (**b**) Superpixel visual maps.

The SLIC algorithm first samples K cluster centers at regular intervals, then moves them to the lowest gradient position within the corresponding 3*3 neighborhood. This is performed to avoid placing them on edges and to reduce the chance of selecting noisy pixels. Each pixel in the image is associated with the nearest cluster center overlapping with the search region around that pixel. After all pixels are associated with the nearest cluster center, a new center is computed as the average labxy vector of all pixels belonging to the cluster. Then, we iteratively repeat the process of associating pixels with the nearest cluster center and recalculating the cluster centers until convergence. Figure 2 is the superpixel segmentation image by the SLIC method.

#### 2.1.2. Saliency Measure

Given the dataset $X=\left\{x_{1},\cdots ,x_{l},x_{\left(l+1\right)},\cdots ,x_{n}\right\}\in R^{m\times n}$, some nodes are selected as query nodes and others are sorted according to their relevance to the query nodes. We build a graph G=(V,E), with the node V as the dataset X and the edge E as the affinity matrix $W={\left[w_{ij}\right]}_{n\times n}$, which determines the weight between node *i* and node *j*. We compute the degree matrix $D=diag\left\{d_{11},...,d_{nn}\right\}$ of the graph G, where $d_{ii}=\sum_{j}w_{ij}$. We find the ranking scores of each superpixel by solving this optimization problem:

(1) $$f^{*}=\arg\min_{f}\frac{1}{2}\left(\sum_{i,j=1}^{n}w_{ij}{∥\frac{f_{i}}{\sqrt{d_{ii}}}-\frac{f_{j}}{\sqrt{d_{jj}}}∥}^{2}+\mu \sum_{i=1}^{n}{∥f_{i}-y_{i}∥}^{2}\right)$$

where $y=\left\{y_{1},y_{2},y_{3},...,y_{n}\right\}$, $y_{i}=\left\{\begin{array}{cc} & 1\;\mathrm{if}\;x_{i}=query\\ & 0\;\mathrm{otherwise} \end{array}\right.$, and μ is the balance constant, regulating the interplay between the smoothness constraint (the first term) and the fitting constraint (the second term). Essentially, it controls the degree to which a ranking function maintains consistency between neighboring nodes (smoothness constraint) and its adherence to the initial query assignment (fitting constraint). The optimal solution of *f* is obtained by:

(2) $$f^{*}={\left(D-\alpha W\right)}^{-1}y$$

where α is a balance constant. α = 1/(1 + μ) and *S* is the normalized Laplacian matrix, $S=D^{-1/2}WD^{-1/2}$. The parameter α is empirically chosen, α=0.99.

In this method, we treat the four edges of the image as background query nodes, and other nodes are ranked by their distance from the four background query nodes. Then, the results of the four boundaries are combined to construct the original saliency map. Then, we use the average saliency of the whole initial saliency map as a dynamic threshold to segment the original saliency map, and the final saliency map is produced.

### 2.2. Dnn-Based Method

The use of deep neural networks (DNNs) has enabled remarkable advances in machine learning in the past few years. The success of DNN mainly comes from extracting the deep features of images from large training datasets to construct deep structures. Binary classification is a method of computing saliency values based on superpixels. Li and Yu [18] extracted multi-scale image regions for each superpixel, input them into a multi-scale CNN, and combined their features to compute the saliency of each superpixel. Wang et al. [19] used prior information to continuously introduce the prediction results of the previous level to correct the current detection results through a recursive FCN. In order to efficiently identify salient objects, Zhao et al. [20] suggested a multi-context information learning network that integrates local and global context information. It is noticed that the saliency detection model based on DNN gradually transitions from a superpixel-wise region prediction model to the pixel-wise model. This means that the saliency of each pixel in the entire image is directly predicted by the FCN. Subsequently, researchers began to dig deeper into the pixel-wise saliency detection task. In order to accomplish saliency detection, Hou et al. [21] employed edge information for multi-scale feature fusion. A pooling-based approach was presented by Liu et al. [22] in order to acquire multi-level feature fusion and spatial context information.

We apply multiple levels of features to detect saliency objects, not only on the superpixel level, but also on the pixel level. Pixel-wise feature learning is the first step, which is to directly output an initial saliency estimation image based on the pixel-wise DNN model. The second and third steps are superpixel-wise feature learning, which exploit the superpixel-wise model to produce coarse saliency maps and final saliency maps, respectively. We leverage the DNN model’s pixel-wise and superpixel-wise classification capabilities to increase the precision and robustness of our method.

## 3. The Proposed Algorithm

**Figure 3:**
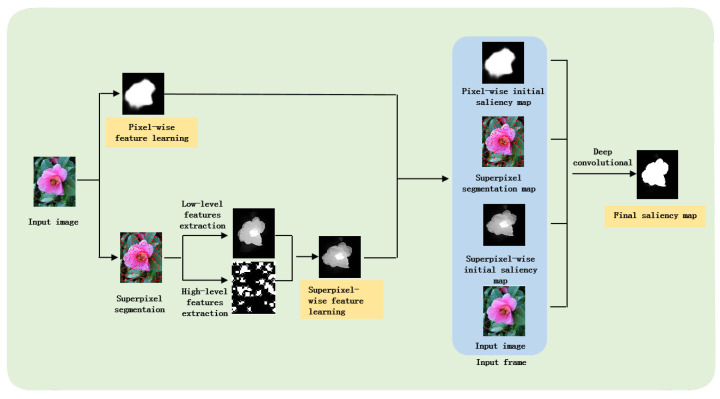
Overall pipeline of our method. The three processing steps are highlighted in orange color. First, an input image is given to the pixel-based and superpixel-based feature learning steps, respectively. The generated two initial saliency maps and the original RGB input image, along with the superpixel segmentation map generated by the SLIC algorithm, form a six-dimensional indicator vector, from which the final saliency map is generated.

There are the following three steps in our process: pixel-wise feature learning, superpixel-wise feature learning, and superpixel-wise feature learning for final saliency maps. Figure 3 shows the flow chart of our method.

### 3.1. Feature Learning Based on Pixel-Wise

**Figure 4:**
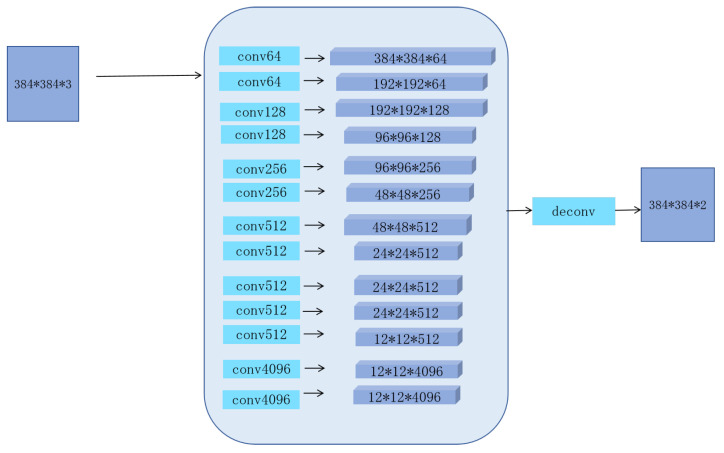
The architecture of our DNN network.

**Figure 5:**
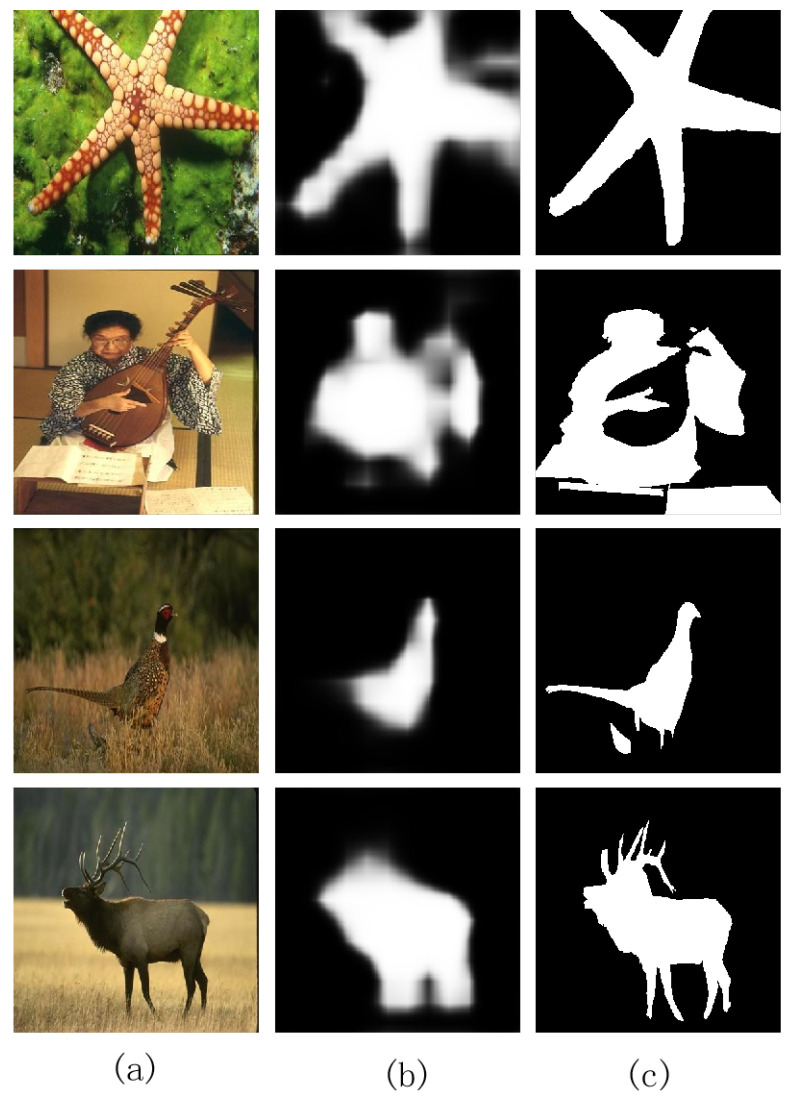
Output cases of the feature learning based on pixels. (**a**) Input images. (**b**) Saliency maps of the feature learning based on pixels. (**c**) Ground truth.

Pixel-wise feature learning is a pixel classification method that assigns a label to each image pixel, indicating its likelihood of being part of the foreground or background. In [14,23], pixel-wise prediction has achieved a dramatic improvement in semantic segmentation. We apply a classification model based on DNN. The network architecture is detailed in Figure 4. The model fixes the size of the initial image to 384×384. If so, please revise. 384, and the last few convolution layers are converted into 1×1. The heatmaps of foreground and background are directly produced at the conv8 layer. Then, we upsample the heatmaps to 224×224 by applying the bilinear interpolation. The network has been trained in the dataset DUT-OMRON. We can apply the model to obtain the coarse semantic segmentation map. In Figure 5, we show the visual outputs of the results after the step. From Figure 5, pixel-wise feature learning can accurately distinguish the salient objects from the background, but the salient region is not uniformly highlighted.

### 3.2. Feature Learning Based on Superpixels

To obtain the coarse contours of the salient objects, we apply feature learning based on superpixels. This method produces an initial saliency estimation for each superpixel using both hand-crafted and deep features. The deep features, which are rich in semantic information, can effectively distinguish the objects from the background, but they are not precise enough to locate the object boundaries. The low-level features, which consist of various hand-crafted features, can measure the similarity among superpixels with a strong discriminative function. Therefore, we combine these complementary features for a better result. We also resize the initial image to 224×224, and then segment it into several superpixels.

#### 3.2.1. Low-Level Appearance Features

To represent each superpixel, we extract five kinds of low-level features related to color and texture, which are common descriptors of the surface property in an image. For color feature, the average value (3 features) and the proportional distribution of different colors using a color histogram (32 features) are computed in the CIELab color space. For texture, we use the following three methods: the Gabor filter, the local binary pattern (LBP), and spectral residual. The Gabor filter produces 36 features with 12 orientations and 3 scales. The LBP generates 1 feature by calculating a circular symmetric neighborhood of pixels. The spectral residual also yields 1 feature by applying an inverse transform and a Gaussian smoothing [24]. We combine all these hand-crafted features into a new feature vector for each superpixel.

#### 3.2.2. High-Level Semantic Features

Image variations usually affect low-level features, so hand-crafted features are specific and sensitive, while deep neural networks (DNNs) are capable of capturing the semantic information of an image. We use the VGG-19 net [25], which was trained on the ImageNet dataset [26,27,28], to extract the deep features of each superpixel. For every convolution layer, the pre-trained VGG-19 network generates a set of feature maps. The semantic contrast between the objects and the background increases in deeper layers, while the spatial resolution for object localization improves in earlier layers. For saliency detection, we prioritize the object locations over their semantic category. Therefore, we combine the semantic feature of the deeper layers with the location information of the earlier layers to distinguish the objects from the background. We use the feature maps of conv 1-2, conv 3-4, and conv 4-4 layers to construct a high-level feature vector with dimensions of 64, 256, and 512, respectively.

#### 3.2.3. Manifold-Space Ranking Based on Superpixels

We exploit a ranking method in the manifold space, which is described in Section 2.1, to obtain a binary classification at the superpixel level.

We extract 832 deep features and 73 hand-crafted features. High-level features are rich in semantics and can separate the objects and the background accurately, but they cannot locate the salient objects. Low-level features can recognize the similarity between superpixels with a strong discriminative ability. Therefore, all these 905-dimensional features form a feature vector to describe each superpixel. The feature vector is denoted as $c=(c_{1},c_{2},c_{3},...,c_{905})$. Let $d(c_{i},c_{j})$ denote the Euclidean distance between node *i* and node *j*, which have feature vectors $c_{i}$ and $c_{j}$, respectively. It is given by:

(3) $$d\left(c_{i},c_{j}\right)=∥c_{i}-c_{j}∥,$$

We write $w_{i,j}\left(c\right)$ as the weight between node *i* and node *j*. It is given by:

(4) $$w_{ij}\left(c\right)=e^{\frac{c(n_{i},n_{j})}{\delta^{2}}},$$

where δ serves as a parameter to change the weight’s strength between a pair of nodes. The parameter $\delta^{2}$ is empirically chosen, $\delta^{2}=0.1$.

Then, we divide the saliency detection into two stages. We use the background prior [27,29] in the first part, and select the image boundary region as the background query nodes. We start with the top boundary of the image as the background query nodes. The other nodes calculate their ranking values according to the relevance with the background query nodes using Equation (2). The ranking values indicate the relevance to the background region. We use the complement of the ranking values to indicate the saliency values, and normalize to 0–1. The saliency map using the top boundary query nodes are written as:

(5) $$S_{t}\left(i\right)=1-\bar{f^{*}}\left(i\right),$$

where *i* serves as the node on graph *G*. $\bar{f^{*}}\left(i\right)$ denotes the normalized vector. We also use the bottom, left, and right boundaries as query nodes, and calculate the saliency maps $S_{b}$, $S_{l}$, and $S_{r}$. Then, we combine these four saliency maps through the following process:

(6) $$S_{bg}\left(i\right)=S_{t}\left(i\right)*S_{b}\left(i\right)*S_{l}\left(i\right)*S_{r}\left(i\right),$$

We compute the saliency of each node to obtain the saliency map of the first stage. We segment with an adaptive threshold segmentation method, and obtain the foreground seed nodes, which are the query objects of the second stage. The saliency map based on superpixels is obtained as follows:

(7) $$S_{fg}\left(i\right)=\bar{f^{*}}\left(i\right)\;\;\;\;\;\;\;\;\;\;\;\;\;\;\;\;i=1,2,\ldots \ldots N,$$

where *i* serves as the node on graph *G*. $\bar{f^{*}}\left(i\right)$ is the normalized vector.

### 3.3. Feature Learning for Final Saliency Maps

**Figure 6:**
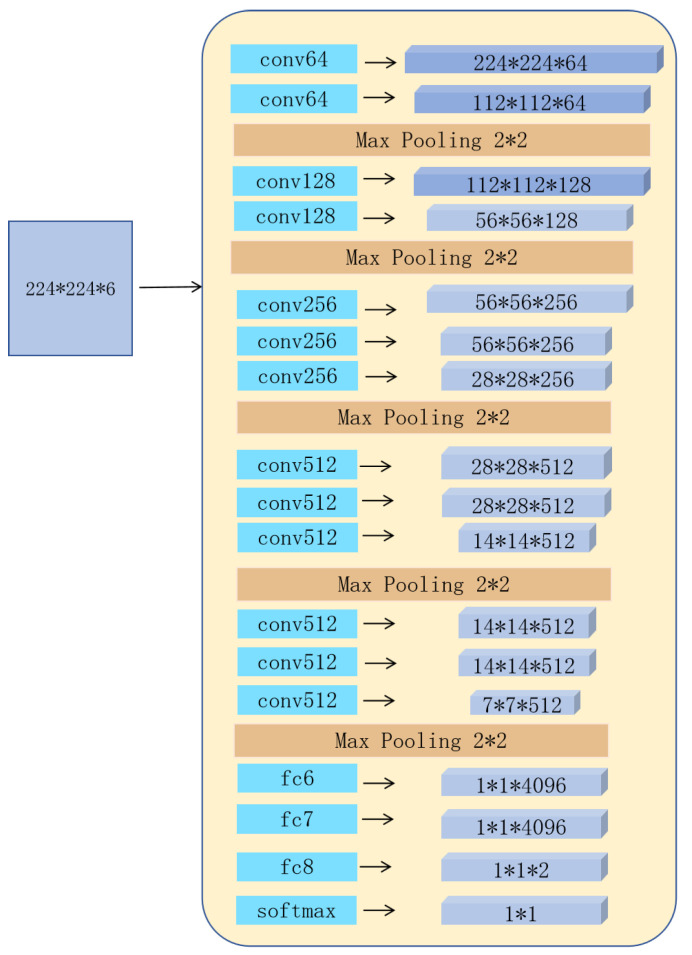
The architecture of our VGG-16 network.

After the feature learning based on pixels and superpixels, we obtain two coarse saliency maps, respectively. In this part, we adopt a deep convolutional network to generate the final saliency map. Considering the comprehensive efficacy, we use a pre-trained VGG-16 as our basic network model. Network architecture is detailed in Figure 6. The key difference between our architecture and DNN lies in the input images, which is a six-dimensional dictator image with 224×224. The input images includes: the original RGB image, which is a three-dimension feature image, the two saliency maps based on pixels and superpixels, and the superpixel-segmented original image. The superpixel-segmented image is generated using the SLIC method (see Section 2.1.1). Specifically, we select the superpixel to be classified and mark it on a 224×224 background. Next, we assign the maximum intensity to the pixels within the selected superpixel, while keeping all other pixels at zero intensity. Our network model can estimate the location of the salient regions with the two coarse saliency maps, while the superpixel indication image can accurately mark the superpixel for classification.

**Figure 7:**
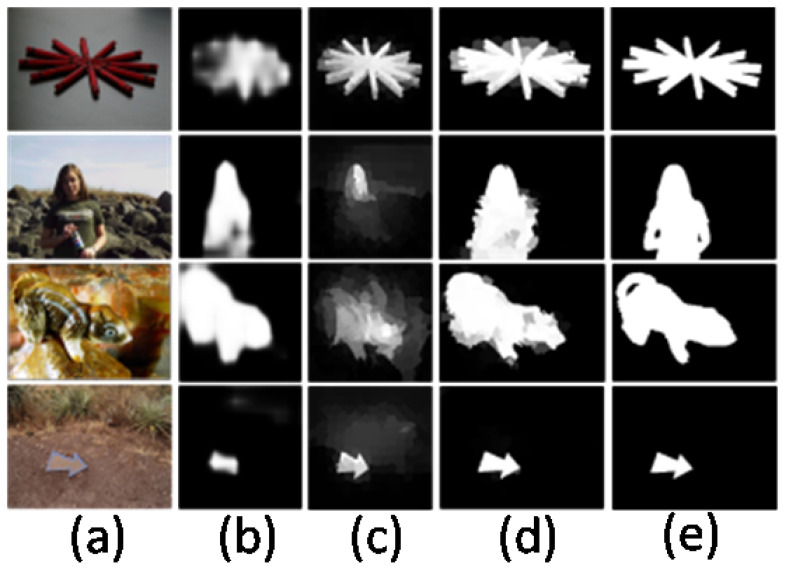
Example outputs of the pixel-wise, superpixel-wise, and the final saliency map. (**a**) Input images. (**b**) Feature maps based on pixels. (**c**) Saliency maps based on superpixels. (**d**) Final saliency maps. (**e**) Ground truth. Note that feature learning based on pixel-wise and superpixel-wise feature learning provides complementary contributions to the final saliency map, which is the result of the proposed method.

Figure 7 displays the saliency maps that resulted from the three processes. It is evident that the saliency maps from the first two steps cannot accurately segment and highlight the salient objects, but these two steps have a complementary effect on the results of the third step. Therefore, the combination of pixel-wise and superpixel-wise saliency maps significantly improves the overall robustness of our method.

## 4. Experiments

### 4.1. Datasets

We evaluate our method’s performance on five classic datasets. The datasets are HKU-IS [7], ECSSD [30], PASCAL-S [18], SOD [31], and DUT-OMRON [32]. There are 4447 images with different prominent objects in the HKU-IS dataset, many of which are discontinuous and have low contrast with the background. There are 1000 images with intricate and rich content in the ECSSD database. The PASCAL-S dataset has 850 images with highly challenging backgrounds. The SOD dataset contains only 300 images, but it is more complex. Some images contain more objects, and the differences between the objects are strong, sometimes the objects are mixed with the background, and it is recognized as very challenging [11]. In total, 5168 high-quality images are included in the DUT-OMRON dataset, with both complex backgrounds and objects. The ground truth images from all five datasets are used to assess the saliency map’s performance.

### 4.2. Evaluation Metrics

As our evaluation measures, we use the precision–recall (PR) curve, the receiver operating characteristic curve (ROC), the F-measure, and the mean absolute error (MAE).

In order to obtain a PR curve, a binary image is first segmented using an integer threshold ranging from 0 to 255. The binary image created using various thresholds is then compared to the ground truth image to obtain a PR curve.

For each threshold changing between 0 and 255, the binarized map obtained by different thresholds is compared with ground truth image in the false positive rate (FPR) and the true positive rate (TPR). The FPR and TPR construct the ROC curve.

The recall and precision measurements are assessed using the F-measure, and is another important performance evaluation indicator. Its calculation formula is:

(8) $$F_{\beta }=\frac{(1+\beta^{2})Precision\times Recall}{\beta^{2}Precision+Recall},$$

where β is a parameter. According to [33], β is set to 0.3.

Additionally, the saliency detection results are assessed using the MAE, which solely compares the saliency map with the ground truth image without the need for binarization or segmentation. The detection result is more similar to the ground truth image when the MAE is less. The equation is:

(9) $$MAE=\frac{1}{N}\sum_{N}^{i-1}\left|S(i)-G(i)\right|,$$

where S(i) is the saliency map, and the G(i) is the ground truth map.

We test these methods on the same software and hardware configuration.

### 4.3. Comparision with State-of-the-Art Methods

We compare our proposed method with seven state-of-the-art conventional (nonlearning based) saliency detection methods and seven learning-based methods. The seven conventional methods are FT [10], RC [33], RR [34], GR [35], MSS [36], MR [12], and LHMR [37]. The seven learning-based methods are DRFI [38], HCA [22], HDCT [39], LEGS [40], MCDL [20], SSD-HS [41], and ELD [42]. The results of various saliency detection methods on the five datasets are shown in Table 1. Due to the training and learning process of the high-level features, the overall performance of the learning-based methods is much better than that of the conventional methods on the five datasets. In Table 1, our proposed method has the better results than all of the 14 state-of-the-art methods. On the HKU-IS, ECSSD, and PASCAL-S datasets, the recall evaluation metrics of the proposed method are lower than that of the ELD method, but the precision of the proposed method is 0.0382, 0.0421, and 0.0239 higher than that of the ELD method, respectively, and F-measure of the proposed method also is 0.0284, 0.0291, and 0.0182 higher than that of the ELD method on the three datasets, respectively. Therefore, considering all datasets and evaluation metrics, the proposed method is obviously better than the comparison methods and is more robust.

**Figure 8:**
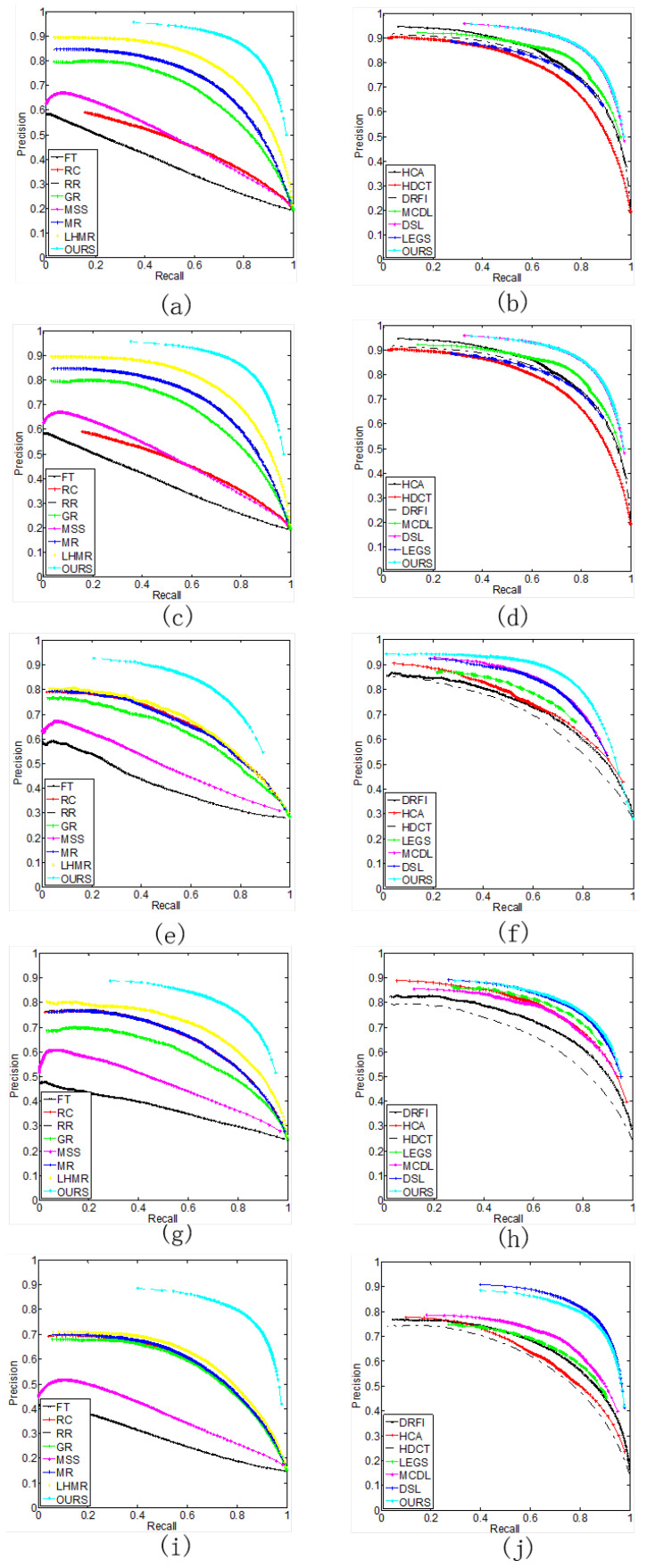
The five datasets’ precision–recall curve for our methods and compared methods. (**a**,**b**) HKU-IS dataset. (**c**,**d**) ECSSD dataset. (**e**,**f**) SOD dataset. (**g**,**h**) PASCAL-S dataset. (**i**,**j**) DUT-OMRON dataset.

**Figure 9:**
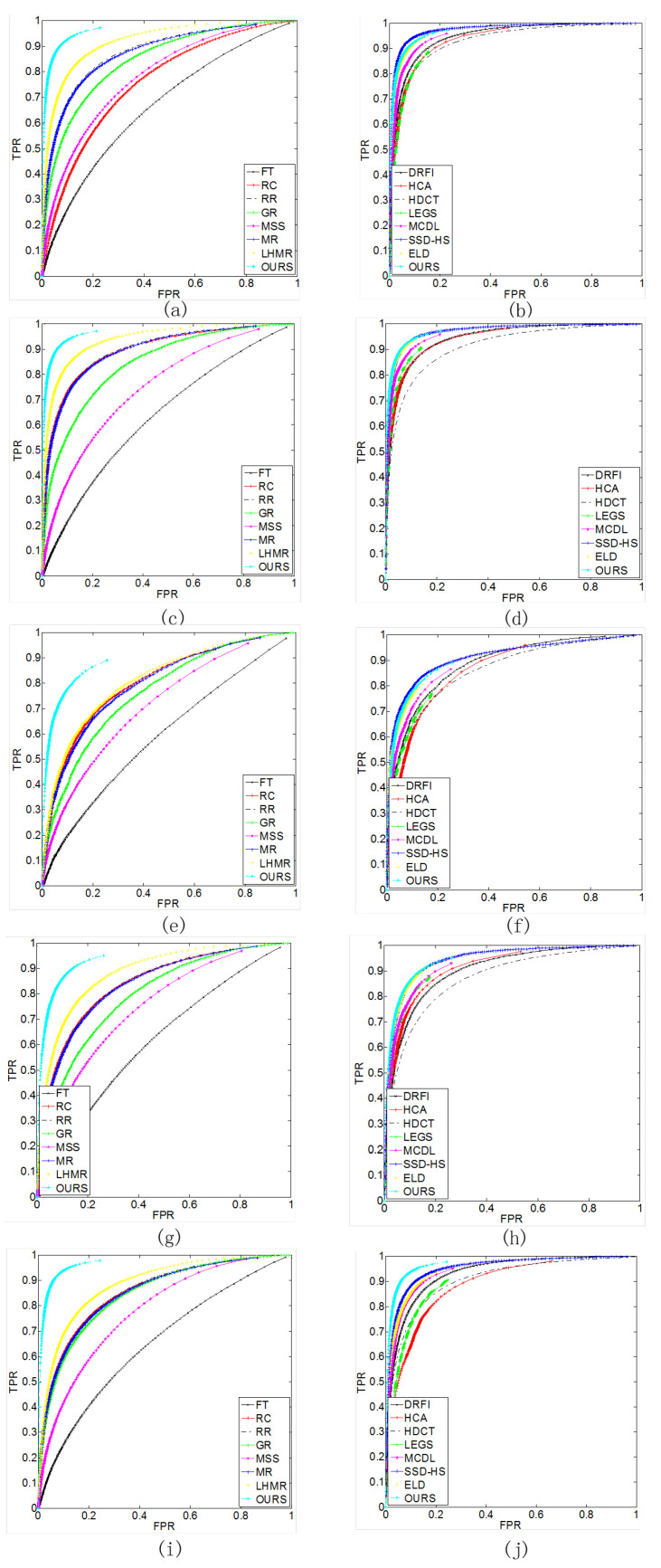
The five datasets’ roc curve for our methods and compared methods. (**a**,**b**) HKU-IS dataset. (**c**,**d**) ECSSD dataset. (**e**,**f**) SOD dataset. (**g**,**h**) PASCAL-S dataset. (**i**,**j**) DUT-OMRON dataset.

In addition, the proposed method also compares with other methods on the PR curves, the ROC curves, the F-measure curves, and the saliency maps of the detection results on the five datasets, which measure the performance of the different models from both objective evaluation metrics and subjective perception. Figure 8 and Figure 9 show the PR curves and ROC curves. On the ECSSD, PASCAL-S, SOD, and DUT-OMRON datasets, the PR curves and ROC curves of the proposed method are the highest, indicating that the detection performance of the proposed method is better than the other methods. On the HKU-IS dataset, the proposed method also outperforms most of the comparison methods in detection performance.

**Figure 10:**
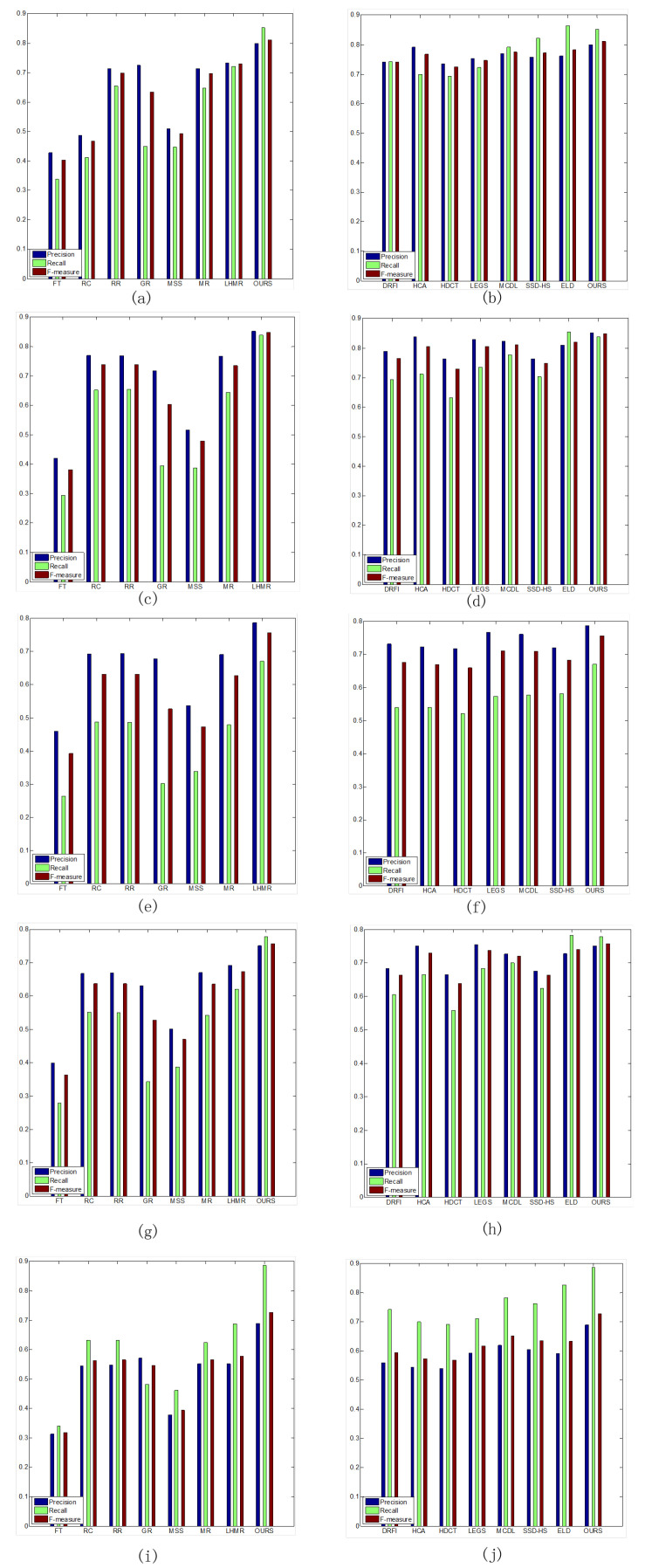
The average precision, recall, and F-measure of our method and state-of-the art methods on the five datasets. (**a**,**b**) HKU-IS dataset. (**c**,**d**) ECSSD dataset. (**e**,**f**) SOD dataset. (**g**,**h**) PASCAL-S dataset. (**i**,**j**) DUT-OMRON dataset.

We also compare the F-measure curves, as shown in Figure 10. The F-measure is the harmonic mean of precision and recall, which reflects the comprehensive indicator of saliency detection. From Figure 10, it can be seen that the proposed method obtains the best performance on the five datasets.

**Figure 11:**
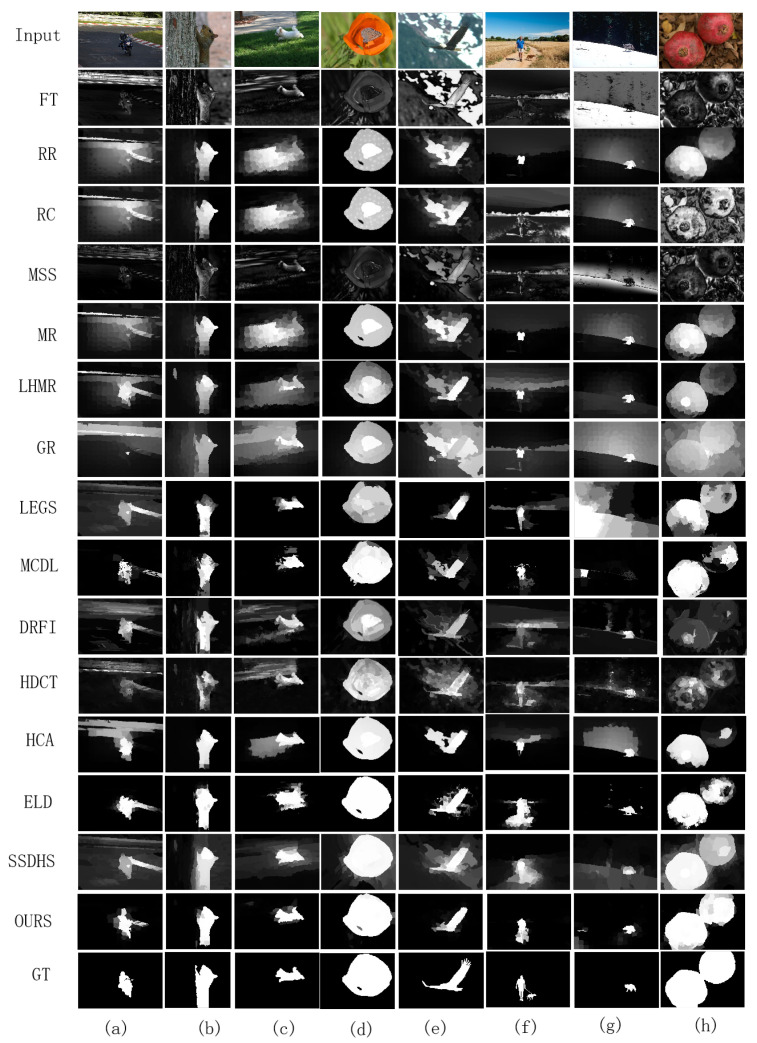
Saliency maps of the proposed method and against methods on five datasets. (**a**,**b**) The images with low contrast. (**c**–**e**) The images with complex background. (**f**,**g**) The images with small objects. (**h**) The image with two objects.

Figure 11 shows the vision comparison of the saliency maps detected by the proposed method and the other methods. It can be seen that, in low-contrast images (cols 1–2), conventional methods fail to detect the complete salient objects in the image (col 1), and the detected salient objects in the image (col 2) were also not smooth. Although deep learning-based methods can detect the salient object region more accurately, the detected object region and background boundary are not sharp except for the proposed method. The proposed method can accurately segment the object region and the background boundary. For complex background cases (cols 3–5), the proposed method can also detect the object region completely and accurately, and the detection results are smooth and uniform, while most other methods have poor detection performance and background noise. For images with small salient objects (cols 6–7), neither traditional methods nor deep learning-based methods can detect salient objects well, some methods even fail to detect salient regions. But compared with other methods, the proposed method detects more complete salient objects and suppresses background noise. In images with multiple objects (col 8), only a few methods detect two salient objects, our method not only detects two salient objects, but also highlights them. In general, the saliency results of proposed method has a clear texture, an obvious contour, and better visual effects.

### 4.4. Runtime Comparision

To assess the efficiency of our method, we compare it with seven methods, three of them are conventional algorithms and four of them are learning-based algorithms. The seven methods have the higher performance according to the Table 1. The runtime of processing per image in PASCAL dataset is computed on the same PC with Matlab R2016b-64bit, and the results are shown in Table 2. This table shows that our method has a faster runtime than the other learning-based methods and also achieves a comparable performance with the conventional algorithms.

## 5. Limitation

**Figure 12:**
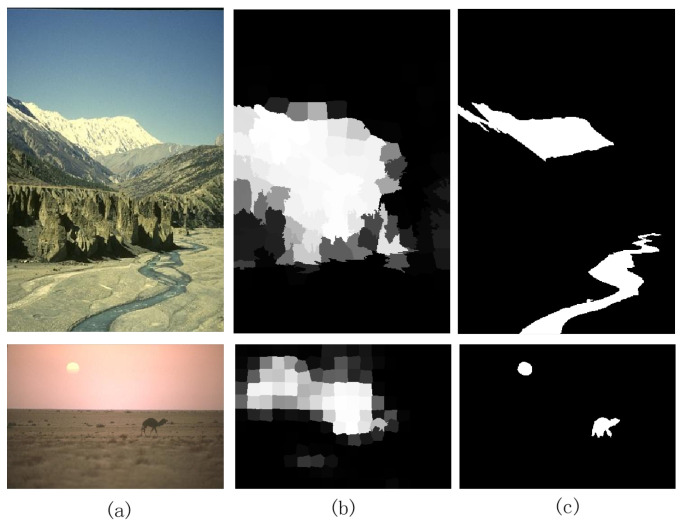
Failed cases of our method. (**a**) Input images. (**b**) Our method. (**c**) Ground truth.

Our model uses multiple-level feature learning to detect salient objects. From the experiments above, it can be observed that our algorithm can accurately detect all the objects when the objects appear at the image’s edge. However, we also find that our algorithm fails to detect the salient objects when these salient objects appear at the image’s edges and have a low color contrast with the image boundary regions. The failure cases are shown in Figure 12. Since our algorithm uses the background prior in the manifold ranking stage, the background prior regards the four boundary regions of an image as background. If the salient objects appear in the boundary region while having a low color contrast with the background, our algorithm regards the objects as the background. How to train our model to detect the object correctly is our future research content when objects exist in the boundary region with a low-contrast image. These images are also a challenge for the other against methods.

## 6. Conclusions

In this work, we propose a saliency detection method via multiple-level feature learning. First, we use a DNN model to generate a pixel-wise coarse saliency map. Second, we extract the low-level and high-level features of each superpixel, and then form a new feature vector. We use a manifold ranking algorithm to assign a saliency value to each superpixel and form our superpixel-wise saliency map. Finally, we put the two coarse saliency maps obtained in the previous two steps, the initial RGB image and the superpixel segmentation image, into a pre-trained DNN model to obtain an accurate final saliency map. From the perspective of qualitative evaluation and quantitative evaluation, our method has significant improvement over 14 comparison algorithms on five public datasets. In the next step of work, we will expand the scope of the training set to make the model more robust.

## Figures and Tables

**Table 1:** Results of our method’s quantitative assessment in comparison to 14 other methods.

Dataset	Metric	FT	GR	RR	RC	MSS	MR	LHMR	DRFI	HCA	HDCT	LEGS	MCDL	SSD-HS	ELD	OURS
HKU-IS	P	0.4277	0.7247	0.7129	0.4867	0.5091	0.7134	0.7334	0.7413	0.7910	0.7354	0.7537	0.7702	0.7583	0.7612	** 0.7994**
	R	0.3385	0.4490	0.6551	0.4121	0.4467	0.6470	0.7217	0.7420	0.6999	0.6935	0.7217	0.7917	0.8210	**0.8633**	0.8522
	F	0.4032	0.6348	0.6987	0.4672	0.4932	0.6969	0.7306	0.7414	0.7680	0.7253	0.7463	0.7751	0.7719	0.7826	** 0.8110**
	MAE	0.2525	0.2568	0.1723	0.2923	0.2027	0.1744	0.1644	0.1475	0.1134	0.1645	0.1193	0.0919	0.1771	0.0747	**0.0728**
ECSSD	P	0.4204	0.7177	0.7689	0.7693	0.5157	0.7675	0.7829	0.7896	0.8380	0.7640	0.8290	0.8234	0.7635	0.8104	** 0.8525**
	R	0.2927	0.3932	0.6545	0.6528	0.3866	0.6447	0.6770	0.6942	0.7120	0.6325	0.7355	0.7778	0.7030	**0.8549**	0.8387
	F	0.3820	0.6029	0.7391	0.7389	0.4788	0.7352	0.7556	0.7653	0.8051	0.7290	0.8054	0.7653	0.7487	0.8202	** 0.8493**
	MAE	0.2910	0.2840	0.1854	0.1838	0.2447	0.1875	0.1716	0.1702	0.1192	0.1975	0.1180	0.1007	0.1923	0.0796	**0.0792**
SOD	P	0.4598	0.6780	0.6944	0.6934	0.5376	0.6911	0.7023	0.7312	0.7226	0.7176	0.7673	0.7617	0.7212	0.7762	** 0.7868**
	R	0.2642	0.3018	0.4860	0.4875	0.3386	0.4792	0.4925	0.5391	0.5388	0.5204	0.5783	0.5772	0.5816	0.6566	** 0.6711**
	F	0.3927	0.5266	0.6319	0.6318	0.4734	0.6271	0.6395	0.6762	0.6699	0.6599	0.7119	0.7094	0.6834	0.7449	** 0.7567**
	MAE	0.3175	0.3172	0.2617	0.2585	0.2807	0.2633	0.2642	0.2276	0.2028	0.2449	0.1973	0.1818	0.2223	0.1581	**0.1578**
PASCAL-S	P	0.3988	0.6310	0.6698	0.6682	0.5014	0.6708	0.6920	0.6842	0.7514	0.6658	**0.7560**	0.7275	0.6764	0.7279	0.7518
	R	0.2789	0.3428	0.5500	0.5512	0.3875	0.5422	0.6212	0.6055	0.6659	0.5592	0.6837	0.7007	0.6239	**0.7827**	0.7795
	F	0.3628	0.5284	0.6377	0.6370	0.4695	0.6360	0.6743	0.6643	0.7298	0.6378	0.7380	0.7212	0.6635	0.7398	** 0.7580**
	MAE	0.3130	0.2994	0.2263	0.2254	0.2501	0.2285	0.2202	0.2071	0.1560	0.2255	0.1486	0.1428	0.2191	**0.1211**	0.1235
DUT-OMRON	P	0.3131	0.5709	0.5486	0.5453	0.3778	0.5512	0.5515	0.5602	0.5438	0.5406	0.5928	0.6198	0.6045	0.5918	** 0.6898**
	R	0.3406	0.4815	0.6317	0.6324	0.4621	0.6242	0.6878	0.7425	0.7000	0.6908	0.7117	0.7827	0.7626	0.8256	** 0.8865**
	F	0.3191	0.5474	0.5658	0.5632	0.3944	0.5665	0.5779	0.5938	0.5733	0.5692	0.6166	0.6510	0.6349	0.6332	** 0.7270**
	MAE	0.2477	0.2605	0.1851	0.1830	0.1770	0.1868	0.1864	0.7572	0.1565	0.1628	0.1333	0.0890	0.1930	0.0929	** 0.0659**

**Table 2:** Runtime of each method.

MR	LHMR	RC	HDCT	DRFI	MCDL	LEGS	ELD	OURS
0.265	0.028	0.657	11.856	9.113	6.356	1.75	1.50	1.625

## Data Availability

Data available in a publicly accessible repository that does not issue DOIs publicly available datasets were analyzed in this study.
